# Bio-functionalized magnetic nanoparticles for cost-effective adsorption of U(vi): experimental and theoretical investigation†

**DOI:** 10.1039/d3ra00799e

**Published:** 2023-05-16

**Authors:** Chanchal Das, Narendra Nath Ghosh, Vandana Pulhani, Goutam Biswas, Pallavi Singhal

**Affiliations:** a Department of Chemistry, Cooch Behar Panchanan Barma University Cooch Behar West Bengal India 736101 goutam@cbpbu.ac.in; b Pakuahat A. N. M. High School Malda West Bengal 732138 India ghosh.naren13@gmail.com; c Environmental Monitoring and Assessment Division, Bhabha Atomic Research Centre Mumbai 400085 India psinghal@barc.gov.in pallavisinghal1987@gmail.com 91-22-2550-5313 91-22-2559-2349

## Abstract

U(vi) removal using cost-effective (production cost: $14.03 per kg), biocompatible, and superparamagnetic *Cinnamomum tamala* (CT) leaf extract-coated magnetite nanoparticles (CT@MNPs or CT@Fe_3_O_4_ nanoparticles) from water resources was studied. From pH-dependent experiments, the maximum adsorption efficiency was found to be at pH 8. Isotherm and kinetic studies were performed and found to follow Langmuir isotherm and pseudo-second order kinetics, respectively. The maximum adsorption capacity of CT@MNPs was calculated to be 45.5 mg of U(vi) per g of nanoparticles (NPs). Recyclability studies suggest that over 94% sorption was retained even after four consecutive cycles. The sorption mechanism was explained by the point of the zero-charge experiment and the XPS measurement. Additionally, calculations using density functional theory (DFT) were carried out to support the experimental findings.

## Abstract 2

Here we report bio-functionalized magnetite nanoparticles (MNPs) for the adsorptive removal of U(vi). Over 94% adsorption is still retained ever after 4th cycle. The experimental adsorption was also investigated and supported by DFT calculations.

## Graphical abstract

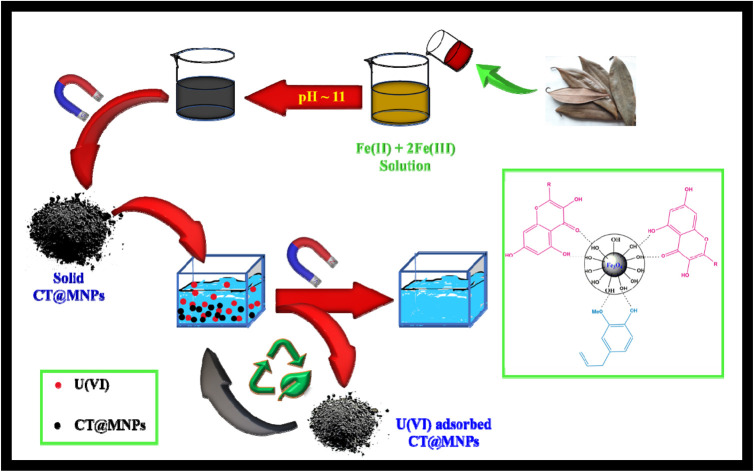

## 1. Introduction

In recent years, wastewater purification has drawn major attention from researchers around the world. To become a part of the industrial revolution, developing and developed countries are upgrading a variety of industries, such as textiles and pharmaceuticals, causing a rise in waste levels within the environment. The increase in waste increases water pollution since the trash from industry is directly discharged into water resources, such as rivers, ponds, and the sea. As a result of water contamination, aquatic life is in peril and at the same time, the quality of drinking water is deteriorating. To overcome issues regarding this, an immediate action is urgently needed. Aside from pollutants, wastewater can contain a variety of hazardous bacteria, such as *E. coli* and *S. aureus*.^1^

Uranium is a well-known radioactive element for nuclear energy as well as a highly chemo-toxic element.^2^ In drinking water, the permissible limit of uranium is 0.03 mg L^−1^ (according to the Bureau of Indian Standards, BIS and World Health Organization, WHO).^3,4^ Uranium is generally present in groundwater as a soluble uranyl (UO_2_^2+^) ion. In addition to the uranyl ion, other forms of uranium, such as UF_6_, UO_2_F_2_, UO_2_(NO_3_)_2_, UO_2_Cl_2_, uranyl acetate, sulphate, and carbonates,^2^ can also exist in groundwater depending on the prevailing conditions. Uranium-contaminated water generally affects the kidney^5^ and therefore suitable techniques, which are low cost,^6^ environmentally friendly,^7^ and efficient,^8^ must be developed to remove U(vi).

**Fig. 1:**
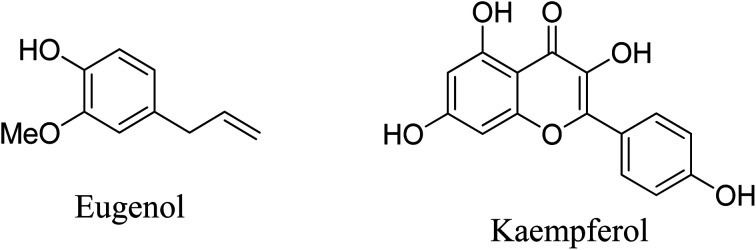
Structure of major components of CT extract.

Various recently-developed materials exhibit high efficiency in U(vi) removal.^9–15^ Nanomaterials are one of the greatest discoveries of all time, with applications in almost every field. Several NPs have been chosen earlier to remove U(vi) from water.^16–19^ Magnetite nanoparticles (MNPs) are unique among nanomaterials because they are superparamagnetic and hence easily separable using a simple static magnetic field. They are also less cytotoxic to both the aquatic and human environments.^20^ Earlier several magnetic composites have been used for uranium removal but only a few report natural products as surface modifiers.^21,22^ MNPs with proper coating can also act as an antifouling agent.^23,24^ It was reported that *A. marina* extract-coated iron NPs act as good antifouling agents.^25^*Cinnamomum tamala* (CT) leaf is the Indian spice, called “Indian bay leaf” or “tejpatta” and is a traditional medicine used in the treatment of scabies, anal diseases, rectum, heart troubles, bad taste, ozena, diarrhea, *etc.*^26^ Since CT leaf has antioxidant, as well as antibacterial properties,^26,27^ it might also be used as an antifouling agent for ships and boats in marine and freshwater system, in future.^28,29^ The CT leaf extract (aqueous) contains kaempferol and eugenol (as the main ingredient) and many flavonoids,^30,31^ which contain various coordinating groups, such as hydroxyl (–OH) and carbonyl ( <svg xmlns="http://www.w3.org/2000/svg" version="1.0" width="10.400000pt" height="16.000000pt" viewBox="0 0 10.400000 16.000000" preserveAspectRatio="xMidYMid meet"><metadata> Created by potrace 1.16, written by Peter Selinger 2001-2019 </metadata><g transform="translate(1.000000,15.000000) scale(0.011667,-0.011667)" fill="currentColor" stroke="none"><path d="M80 1160 l0 -40 40 0 40 0 0 -40 0 -40 40 0 40 0 0 -40 0 -40 40 0 40 0 0 -40 0 -40 40 0 40 0 0 -40 0 -40 40 0 40 0 0 -40 0 -40 40 0 40 0 0 -40 0 -40 40 0 40 0 0 80 0 80 -40 0 -40 0 0 40 0 40 -40 0 -40 0 0 40 0 40 -40 0 -40 0 0 40 0 40 -40 0 -40 0 0 40 0 40 -40 0 -40 0 0 40 0 40 -80 0 -80 0 0 -40z M560 520 l0 -40 -40 0 -40 0 0 -40 0 -40 -40 0 -40 0 0 -40 0 -40 -40 0 -40 0 0 -40 0 -40 -40 0 -40 0 0 -40 0 -40 -40 0 -40 0 0 -40 0 -40 -40 0 -40 0 0 -40 0 -40 80 0 80 0 0 40 0 40 40 0 40 0 0 40 0 40 40 0 40 0 0 40 0 40 40 0 40 0 0 40 0 40 40 0 40 0 0 40 0 40 40 0 40 0 0 80 0 80 -40 0 -40 0 0 -40z"/></g></svg> C=O), hence this extract was used as a surface modifier in nanoparticle synthesis.^32^ The CT extract was also employed for reducing metal ions to produce metal nanoparticles.^33^Fig. 1 shows the structure of the major components of CT extract.

In this study, we have synthesized and characterized eco-friendly CT extract-coated MNPs (CT@MNPs) and used them for the removal of U(vi) from contaminated water. To the best of our knowledge, this is the first time CT-coated MNPs have been explored for U(vi) removal. Because CT@MNPs are superparamagnetic and insoluble, they may be easily separated following wastewater treatment. Furthermore, they are nontoxic,^29^ making them preferable to other CT@NPs.^30,32–35^

## 2. Experimental

### 2.1 Chemicals and instruments

All chemicals were analytical grade and were purchased either from Sigma-Aldrich or a local manufacturer. The simulated uranium-contaminated wastewater was prepared by dissolving uranyl nitrate [UO_2_(NO_3_)_2_·*n*H_2_O] in MilliQ water. CT leaves were collected from the local area of Cooch Behar Panchanan Barma University, Cooch Behar. MilliQ water was used to prepare all aqueous solutions. Various instruments were used for performing different experiments including an ultra-sonicator (Qsonica Sonicator); pH meter (Fisher Scientific, Accumet, Model AB 250); MilliQ plant from Labconco, Water Pro/Ro. The experimental data, *viz.* hydrodynamic size, and zeta potential analysis were obtained using a dynamic light scattering (DLS) instrument obtained from Anton Paar Litesizer 500; Fourier-transform infrared (FTIR) spectra were measured on a Thermo Scientific Nicolet IS50 instrument; powder X-ray diffractometry (pXRD) patterns were obtained on a Rigaku Ultima IV X-ray diffractometer; magnetization studies were performed on an alternating gradient field magnetometer (AGM), PMC MicroMag 2900 Series; high-resolution transmission electron microscope (TEM) images were obtained on a Tecnai G2 instrument, and thermogravimetric analysis (TGA) were performed on an SDT650 instrument.

### 2.2 Methods

#### 2.2.1 Preparation of CT leaf extract

Approximately, 40 g of the CT leaves were dried and cleaned, then crushed to powder and refluxed with 140 mL of MilliQ water. The extract was cooled and stored at 0–4 °C.^30^

#### 2.2.2 Preparation of bare and CT-coated magnetite nanoparticles

The synthesis of bare Fe_3_O_4_ NPs was performed utilizing the technique reported by Singhal *et al.*^22^ To synthesize CT@MNPs, the method reported by Das *et al.* was followed.^29^ Briefly, ferrous chloride (FeCl_2_·*x*H_2_O, 1.015 g) and ferric chloride (anh. FeCl_3_, 2.595 g) were mixed in 80 mL of MilliQ water and stirred to dissolve. About 20 mL of aqueous CT extract was then added followed by the addition of NaOH solution, to maintain the solution's pH at 11. With the formation of NPs, the color of the solution changed to black. The resulting mixture was stirred for ∼60 minutes, and then separated by a powerful magnet (5000 G). The NPs were washed four times using MilliQ water to remove unreacted ferrous and ferric ions and then dried in a vacuum oven generating ∼2 g of the particles. Different characterization methods were adapted to characterize the synthesized NPs, such as pXRD, TEM, FTIR, TGA, DLS, AGM, BET, CHNS, and, zeta potential studies.

Adsorption studies were performed using the previous procedure reported by Singhal *et al.*^22^ A batch method was applied to study the adsorption of U(vi) (having various concentrations: 1, 5, 10, 50, 75 and 100 mg L^−1^) onto 1 mg of CT@MNPs in 1 mL aqueous media, at pH 8 and 298 K temperature. Various pH conditions (pH = 1–10) were applied for the 0.6 mg L^−1^ solution of U(vi) ions. Sonication for 480 min was performed every time except for the contact time experiment. The percentage of the removal was determined by the equation:Removal (%) = (*C*_o_ − *C*_*t*_)/*C*_o_where, *C*_o_ and *C*_*t*_ ( in mg L^−1^) are the concentrations of U(vi) initially and after a certain time, *t* (in min).

Laser fluorimetry (LF 003 uranium analyzer fabricated by Laser Applications and Electronics Division, RRCAT, Department of Atomic Energy, Indore, India) was used to determine the amount of uranium in the supernatant.

## 3. Results and discussion

### 3.1 Characterization of CT-functionalized Fe_3_O_4_ NPs

**Fig. 2:**
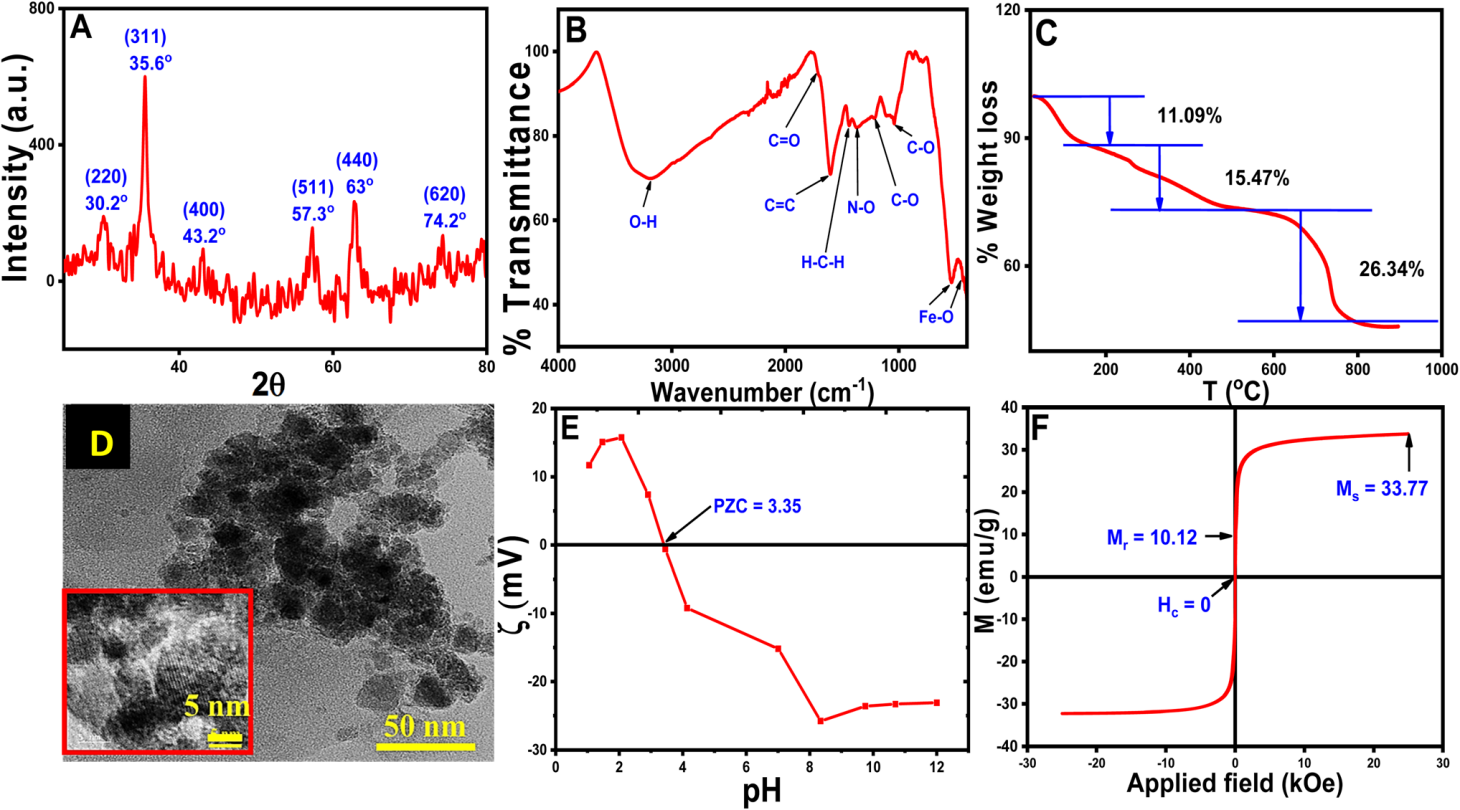
Analytical measurements of CT@MNPs (A) pXRD, (B) FTIR spectra, (C) TG analysis (D) TEM image (resolution: 50 nm), inset: HRTEM image (resolution: 5 nm), (E) zeta potential, (F) magnetic measurements.

The pXRD pattern of the CT-functionalized Fe_3_O_4_ NPs is shown in Fig. 2A. The calculated lattice parameter of the synthesized NPs is *a* = 8.3642 Å having a cubic lattice structure, which closely resembled the lattice parameter for Fe_3_O_4_ (8.397 Å JCPDS no. 19-0629).^36^ For confirmation of CT coating on MNPs, FTIR studies were performed and are shown in Fig. 2B. FTIR peak analysis revealed broadband at 3202.8 cm^−1^ and was attributed to the O–H stretching from eugenol and kaempferol-OH contained in the aqueous CT leaf extract. The other important bands are at 1711.5 cm^−1^ (for C=O stretching), 1601.3 cm^−1^ (for C=C stretching vibration), 1440.2 cm^−1^ (for H–C–H scissoring vibration of –CH_2_– group), 1370 cm^−1^ (for N–O bending) 1212.7 cm^−1^ (for C–O asymmetric stretching vibration of cyclic polyphenols) and 1036.1 cm^−1^ (for C–O stretching vibration) matched well with the reported CT extract and the production of CT extract-coated MNPs was verified from IR data.^30,32,34,37^ Another dual vibration mode appeared at 538 cm^−1^ (tetrahedral Fe–O stretching) and 451.5 cm^−1^ (octahedral Fe–O stretching), which were assigned for Fe_3_O_4_-NPs.^38^

TGA was performed on synthesized NPs and is shown in Fig. 2C. TGA results suggested that the degradation occurs in three steps at variable temperatures. Water molecules, OH^−^ and volatile impurities first leave the surface of the CT@MNPs at a temperature below 180 °C, which appeared with ∼11.09% weight loss.^39^ In the next step (between 180–500 °C), the weight loss was ∼15.47%, due to the decomposition of the coated natural products from the surface of CT@MNP. Another significant weight loss (∼26.34%) was observed between 500–900 °C, which might be attributed to the complete breakdown of the coated natural products into volatile substances.^39–41^ These results proved that the NPs have been successfully coated with organic compounds and also confirm the stability of the NPs.

HRTEM studies were conducted to determine the structure and size of CT@MNPs (Fig. 2D). From the analysis of HRTEM images, it was concluded that the particles were mostly spherical and discrete in nature. The size distribution of the particle was determined to be ∼12 ± 5 nm, as illustrated in Fig. S1.† The DLS study determined the hydrodynamic size of CT@MNPs was 454.5 nm with a polydispersity index (PDI) = 0.26. The sizes were larger due to the hydrogen bonding between the water molecule and the oxide or oxygen-containing surface groups.^42,43^ The results are shown in Fig. S2.†

Zeta potential experiments were performed to determine the surface charge of the CT@MNPs and are shown in Fig. 2E. From the study, it was found that the point of zero charge is at pH ∼ 3.35, a similar result was reported by Ealias *et al.* for their CT extract stabilized NPs.^30^ This finding indicates that the particle surface has a positive charge below pH ∼ 3.35 and a negative charge above it. Magnetic measurements were carried out to determine the magnetic moment of the particles and the data are shown in Fig. 2F. The magnetic saturation value (*M*_s_) was observed to be 33.8 emu g^−1^. In our earlier investigation, we determined the *M*_s_ value for bare Fe_3_O_4_ NPs as ∼70 emu g^−1^.^22^ It was observed that with the addition of CT extract to MNPs the *M*_s_ value reduced to half, which again confirmed the successful coating of CT extract on MNPs. From magnetic measurements, we determined that the content of CT extract in CT@MNPs was ∼50%. According to CHNS analysis, the synthesized NPs include 21, 2, and 0.2% of C, N, and S, respectively. Similar measurement in CT extract was also carried out and % of C, N, and S was observed to be 51, 7.2 and, 0.5%, respectively.

### 3.2 Batch sorption studies

**Fig. 3:**
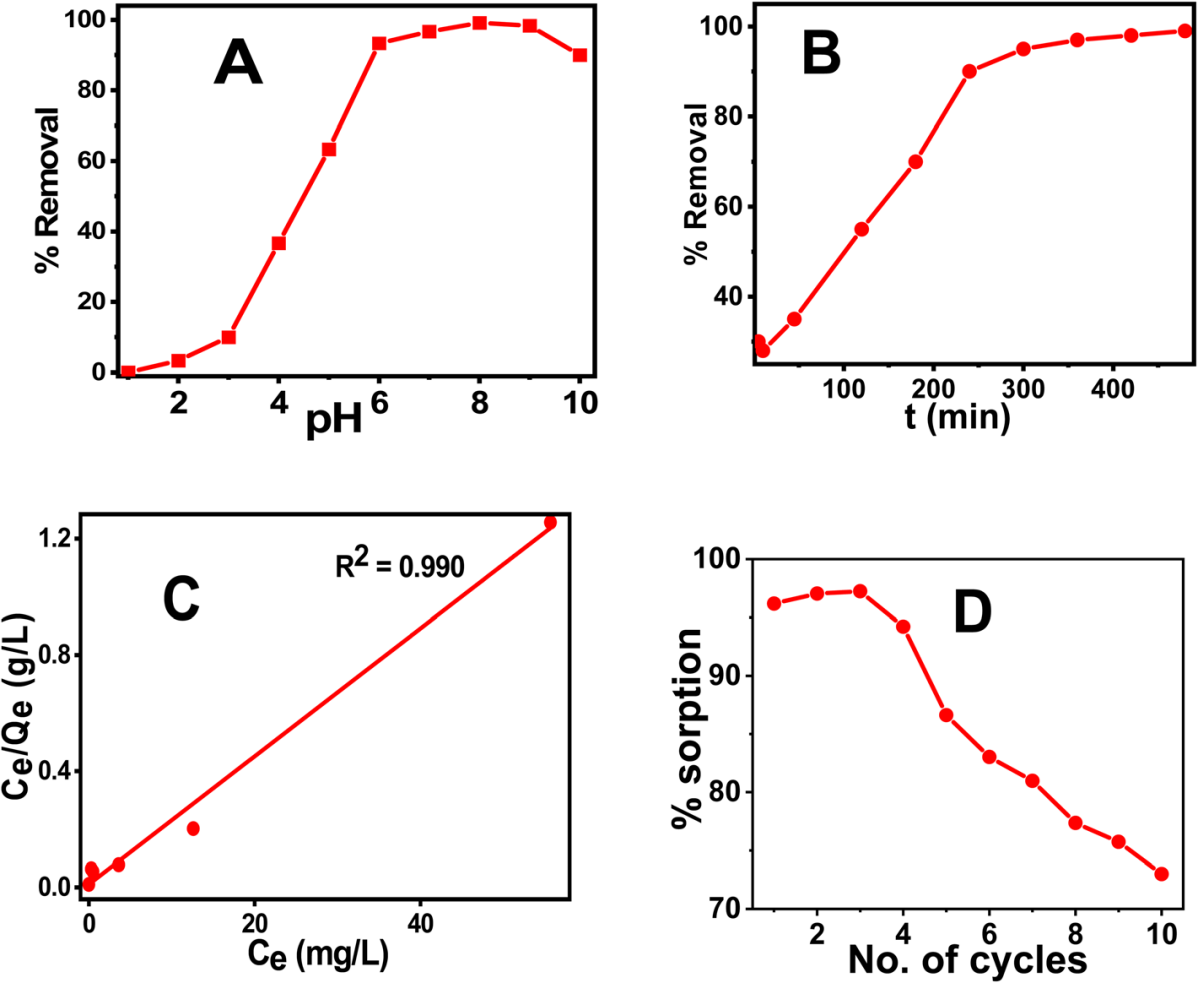
% removal of U(vi) at different (A) pH, (B) time. The initial concentration of U(vi) for the experiment was 1 mg L^−1^ and for the equilibration study pH was maintained at 8. (C) Sorption capacity of U(vi) by fitting the results into Langmuir isotherm with different initial concentrations of U(vi) (1–100 mg L^−1^), (D) % sorption of U(vi) at different cycles. pH-8, equilibration time – 3 h. The *V*/*m* ratio was maintained at 1000 mg L^−1^.

The coprecipitation method was used to prepare CT@MNPs under aerobic conditions at room temperature and in aqueous solutions. Crude extract of CT leaf generally contains kaempferol and eugenol^30,44^ and its quinone form, hence possesses various coordinating groups, *e.g.*, hydroxyl (–OH), methoxy (–OMe), keto (C=O), *etc.* It has been shown that kaempferol and eugenol exist on the surface of CT@MNPs during their preparation by coprecipitation, and these molecules can bind effectively with the uranyl ion. Batch adsorption studies were conducted to check the adsorption behavior of uranyl ions over CT@MNPs. Different pH levels (1–10) were used for the experiments to understand the sorbent's functional pH range, and Fig. 3A displays the findings. From the results, it was evident that >90% U(vi) removal occurred in the pH 6–10 range with the maximum removal at pH 8. It should be noted that the majority of naturally occurring water bodies fall within this pH range. Understanding the interplay between the sorbent and the sorbate is crucial to comprehending this behavior. From zeta potential measurements it is shown that when the pH was increased to >3.5, the surface of the CT@MNPs becomes more negative because of the expulsion of the proton from the NP's surface. At this pH, the prevailing uranium species UO_2_^2+^ interacts with negatively charged surfaces and hence adsorption increases.^45^ The sorption rises with pH increase and peaks at pH 8 because of the interaction among the negatively charged surface and various forms of positively charged uranium species, such as [(UO_2_)_2_(OH)_2_]^2+^, [(UO_2_)(OH)]^+^, [(UO_2_)(OH)_2_], and [(UO_2_)_3_(OH)_5_]^+^.^46^ When pH increases further from 8 to 10%, the removal was decreasing, probably due to the formation of uranium in its negatively charged forms: UO_2_(CO_3_)_2_^2−^ and UO_2_(CO_3_)_3_^4−^.^45,46^ In all further experiments, we kept the solution pH at 8.

Time-dependent sorption measurements were carried out to calculate the equilibration period and data are shown in Fig. 3B. It was observed that after 6 h, the sorption reaches above 90% and on further increase in time the value reaches ∼99%. As a result, the concentration of the dissolved U(vi) after successful adsorption was found to be 0.010 mg L^−1^, which is less than the permissible value of U(vi) concentration according to WHO and BIS guidelines (0.030 mg L^−1^), indicating that the synthesized NPs is effective in removing uranium to a safer level. The results were fitted into various kinetics models and were found to be the best fit for pseudo-second-order suggesting that the sorption process is chemisorption.^47^ Other kinetic data are shown in Fig. S3 and Table S1.†

In order to find the adsorption capacity of the material, studies were carried out with different initial concentrations of U(vi) solutions (1–100 mg L^−1^). The results of the experiment are shown in Fig. 3C and were best fitted in the Langmuir isotherm model. Hence, monolayer adsorption of U(vi) occurs for the CT@MNPs.^48^ The calculated maximal sorption capacity for CT@MNPs is 45.5 mg of U(vi) per g. Other isotherms are plotted in Fig. S4† and the data is given in Table S2.†

We also checked the recyclability of the material and found that even after 10 cycles the material could sorb >70% of U(vi). The experimental findings are shown in Fig. 3D. A desorption study was also performed to validate their reusability. EDTA (ethylene diaminetetraacetic acid), NaHCO_3_ (sodium bicarbonate), Na_2_CO_3_ (sodium carbonate), and HNO_3_ (nitric acid) solutions were chosen for the desorption study. To, 1 mg of NPs containing 28 μg of sorbed uranium 10 mM; 1 mL solutions of each reagent were added. After the combination was sonicated for one hour, the NPs were removed from the solution, and the concentration of the uranium in the solution was determined. EDTA, NaHCO_3_, Na_2_CO_3_, and HNO_3_ were shown to desorb 89, 85, 86, and 83% of the uranium, respectively. This implies that CT@MNPs can be recycled after sorption and that uranium can be removed and used for a variety of purposes. The binding of uranium with the EDTA ligand is a chelating type and EDTA binds very effectively with uranium. CO_3_^2−^ is known as a strong binder of uranium. The addition of HNO_3_ changes the pH of the system and, therefore, uranium at low pH is extracted. Hence, respective reagents were chosen to find better regeneration and reusability results.

Testing the material's selectivity in the presence of several competing ions revealed that it was not selective. It is due to the presence of –OH groups that bind with a majority of the metal ions resulting in non-selective sorption. However, the large sorption capacity and low cost of the material make it a prominent candidate for the purpose of removing harmful ions from wastewater streams. We have also compared the performance of the synthesized sorbent with the earlier discovered material. The comparison is given in Table 1 and it is evident that the material is comparable with the earlier discovered sorbents.

**Table 1:** Comparison of maximum adsorption capacity (*Q*_max_) between CT@MNPs with other commercial/conventional adsorbents towards the removal of U(vi)

Adsorbents	*Q*_max_ (mg g^−1^)	Condition	References
Quercetin@Fe_3_O_4_ NPs	12.33	pH = 3.7, a*t*_e_ ≤ 30 min	49
Fe_3_O_4_ NPs (50–100 nm)	5	pH = 7, *t*_e_ = 360 min	**50**
Humic acid@Fe_3_O_4_ NPs	39.4	pH = 7, *t*_e_ = 60 min	22
SiO_2_@Fe_3_O_4_ NPs	52	pH = 6, *t*_e_ = 180 min	51
Polyoxime@MNPs	141.4	pH = 8, *t*_e_ = 5 min	52
CT@MNPs	45.5	pH = 8, *t*_e_ = 180 min	This study

a *t*_e_ = equilibrium time.

In order to use any material in an industry, it is important that the material fulfill many requirements. The foremost of these are non-toxic, environmentally friendly, low cost, and efficient. To calculate the cost, a complete cost analysis of the synthesized sorbent (Table S3†) was out, and it was observed that the production cost of the sorbent was ∼$14.03 per kg, which is comparatively cheaper compared to other established sorbents (Table 2). In addition to this, Fe_3_O_4_ NPs are completely non-toxic and CT leaves are eco-friendly and are known to have antibacterial properties.^29^ Both properties are essential criteria for any material to become useful for environmental decontamination. Herein the above-mentioned properties of the synthesized sorbent material make them a prominent candidate for U(vi) sorption from waste environmental matrices.

**Table 2:** Production cost comparison between CT@MNPs and other conventional/commercial adsorbents

Metal ion adsorbents	Cost (USD) per kg	References
Chitosan	15.43	53
Activated carbon (industrial grade)	20–22	
Starch xanthates	1.00	
*Citrus sinensis*/ZnO NPs	20.25	54
CT@MNPs	14.03	This study

**Fig. 4:**
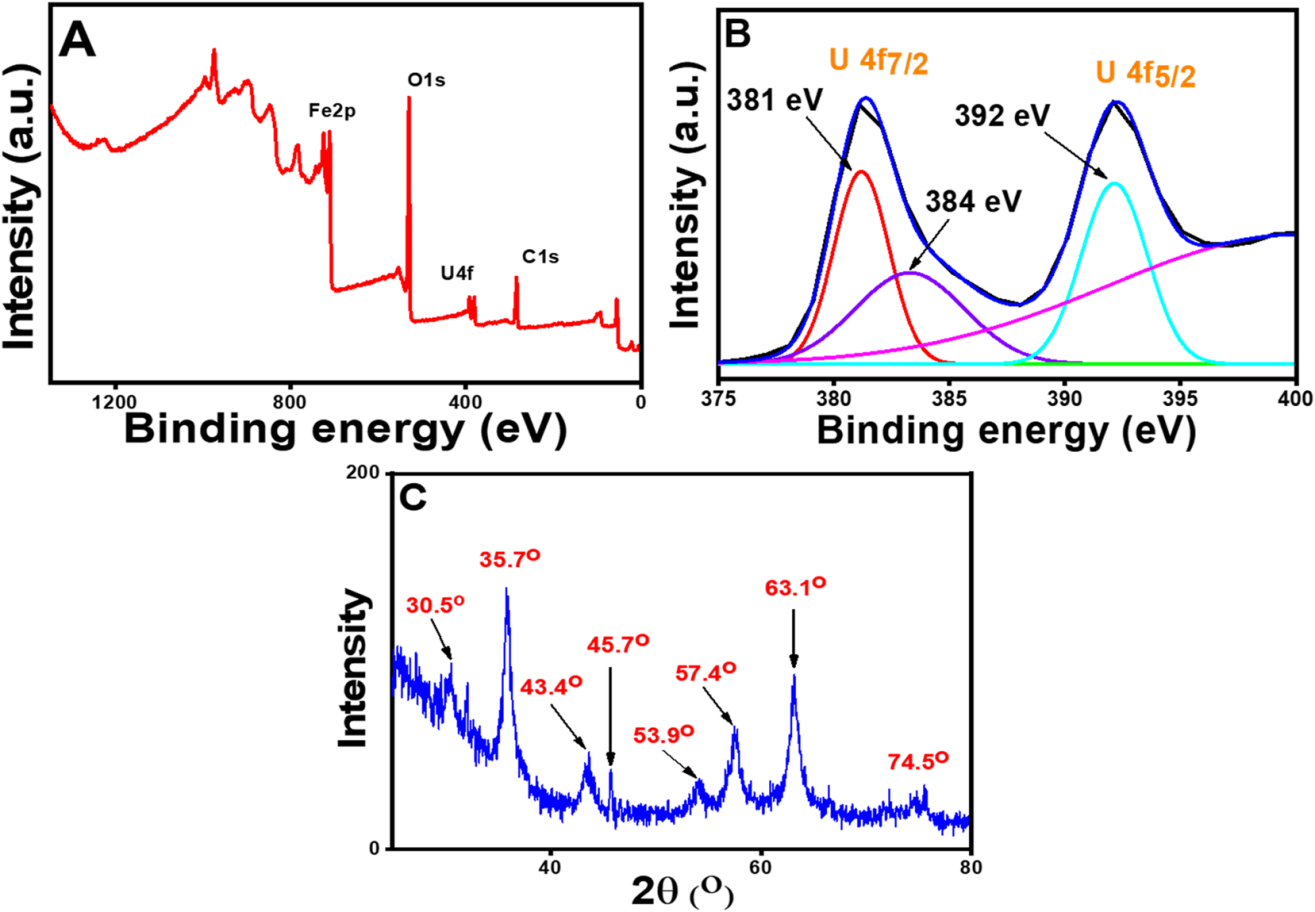
XPS spectra of the U(vi) adsorbed CT@MNPs: (A) wide scanning spectra and (B) high-resolution U 4f spectra and (C) powder XRD patterns of U(vi) adsorbed CT@MNPs.

From the kinetic study, it was found that the adsorption phenomena involved chemisorption, *i.e.*, some type of strong interaction existed between U(vi) species and the nanoparticles. Hence, to establish the mechanism of binding of the U(vi) with the sorbent, XPS and XRD studies were carried out. As the surface of the CT@MNPs was surrounded by various coordinating sites such as –OH, after deprotonation, the surface was completely negatively charged (since the adsorption process was carried out at pH 8, where zeta potential is highly negative), thus it can be concluded that the positively charged uranium ions were electrostatically adsorbed over the surface of CT@MNPs. XPS study (Fig. 4A) revealed that the U(vi) ions were efficiently bound to the CT@MNPs surface by a strong force of attraction (electrostatic attraction). Similar results were reported by Xu *et al.*^52^ and Zhang *et al.*^55^ From Fig. 4B, *i.e.*, on the investigation of high-resolution XPS spectra, two peaks were found: 392.01 eV (U 4f_5/2_), 381.03 eV (U 4f_7/2_), which were also reported by Ouyang *et al.*^56^ and Wang *et al.*^57^ A single contribution of the U(vi) state with binding energies of 392 eV (U 4f_5/2_) and 381 eV (U 4f_7/2_) could be used to match the XPS spectra (Fig. 4B), showing the absence of redox activity during the interaction with polyphenolic compounds.^58^ By comparing Fig. 4C with Fig. 2A, it can be concluded that two new peaks appeared (at 45.7° and 53.9°) when U(vi) was adsorbed on CT@MNPs. These two new peaks arise due to the U(vi) deposition over the nanoparticles.^48,59^

## 4. Density functional theory (DFT) analysis

To investigate the uranyl ion adsorption behavior on CT@MNPs, we have adopted the quantum Espresso's projector augmented wave (PAW) technique^60^ utilizing the PW-SCF code to perform periodic density functional theory (DFT) calculations on the Fe_3_O_4_ (111) surface. In the present study, we have chosen the Fe_3_O_4_ (111) surface since previous studies showed that it has a predominantly naturally-cleaved surface with high thermodynamic stability at ambient conditions.^61,62^ Furthermore, Yu *et al.* within a DFT+*U* scheme showed that compared to the other naturally grown surfaces of Fe_3_O_4_, (111) surface has higher stability.^63^ In addition, the (111) surface is more active for reactions.^64^ As the CT leaf extract is rich in eugenol, we have analyzed its role in the attachment of uranyl to the surface of Fe_3_O_4_ (111) surface with eugenol. At higher pH, different oligomeric species such as (UO_2_)_2_(OH)_2_^2+^, (UO_2_)_3_(OH)_5_^+^, (UO_2_)_4_(OH)_7_^+^, and (UO_2_)_3_(OH)_7_^−^ were found in the solution but all these structures are complicated and quite large. From previous reports, it was found that [UO_2_(H_2_O)_5_]^2+^ is a potential candidate to study the adsorption of organic molecules.^65^ Hence to study the adsorption behavior, in the present work, we have chosen [UO_2_(H_2_O)_5_]^2+^. For optimization, the convergence threshold on the total energy of 10^−6^ (a.u.) and the force of 10^−3^ (a.u.) were set. Wavefunctions were given a kinetic energy cutoff of 64 Ry, while charge density was given a cutoff of 570 Ry. The simulation was conducted utilizing the approximate generalized gradient method (GGA) with Perdew–Burke–Ernzerh (PBE)^66^ and the Marzari–Vanderbilt smearing technique with a smearing threshold of 0.02 and a 1 × 1 × 1 *k*-point mesh.^67^ Additionally in this computation, we used the same lattice parameter as described previously.^68^ The experimental value of 8.394 Å for the standard cell (PBE+*U*) marginally overestimated the lattice parameter (*i.e.*, 8.491 Å) for *U* = 3.5 eV, which is consistent with previously reported data.^68^

To optimize the computational costs, the bottom two atomic layers were kept frozen while the remaining layers were completely relaxed during the calculations.^69^ The lattice cell dimensions were *a* = 18.0121 and *b* = 15.599 Å, including a vacuum zone having a thickness larger than 30 Å in the *c* direction to ensure no contact between the slabs has been optimized and further used for uranyl adsorption calculations.

**Fig. 5:**
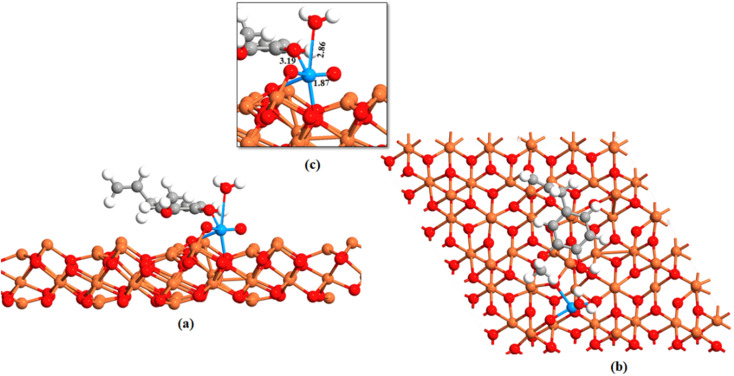
Adsorption of uranyl on the Fe_oct_ terminated Fe_3_O_4_ (111) surface. (a) Side view uranyl, eugenol adsorbed Fe_3_O_4_ (111) surface (b) top view uranyl, eugenol adsorbed Fe_3_O_4_ (111) surface and (c) is the magnification of the attachment. Red, gray, white, and orange color represents oxygen, carbon, hydrogen, and iron atoms respectively.

The ground state energy-minimized geometry of uranyl adsorbed eugenol-modified Fe_oct_ terminated Fe_3_O_4_ (111) surface is illustrated in Fig. 5. Here, we note that the interaction among the uranyl ion and eugenol-modified Fe_3_O_4_ (111) surface happed through the hydroxyl group of eugenol with an O–U bond. U=O bonds for the attached uranyl experience a longer bond length of 1.87 Å than its normal value (1.796 Å).^70^ Previous studies showed that the outer-sphere association of foreign ions with the hexahydrated uranyl ions takes place with a consequent displacement of water molecules coordinated by the uranium until the limiting complex is formed.^71^ As the –O–H bond of the eugenol interacts with U(vi) ion and U=O interacts with the iron atom of Fe_3_O_4_, thereby, U=O bond is longer than its usual value.^70^ These facts essentially illustrate that uranyl adsorption is facilitated by eugenol-modified Fe_3_O_4_ (111) surface.

## 5. Conclusions

In this study, we developed bio-functionalized MNPs to remove uranium(vi) from water. The synthesized NPs have a maximum adsorption capacity of 45.5 mg of U(vi) per g of the sorbent. Kinetic studies suggested that 99% of U(vi) was adsorbed within 8 h. The maximum sorption occurred at pH 8 and the results were explained by the interaction between the sorbent surface and sorbate. The binding of U(vi) over the surface of CT@MNPs was shown using XPS measurements. Furthermore, DFT calculation demonstrates the mode of binding of the uranyl ion and indicates that the adsorption is facilitated by the eugenol-modified Fe_3_O_4_ (111) surface. Both theoretical and experimental results suggest that the NPs are effective for uranium removal.

## Data availability

The data of this study are available upon request from the corresponding author(s).

## Author contributions

Chanchal Das: investigation, formal analysis, and writing—original draft; Narendra Nath Ghosh: theoretical investigation, analysis, and writing draft; Vandana Pulhani; investigation, formal analysis, and writing draft; Goutam Biswas and Pallavi Singhal: methodology, funding acquisition, supervision, and writing—review and editing.

## Conflicts of interest

The author declares that there is no conflict of interest regarding this work.

## Supplementary Material

RA-013-D3RA00799E-s001
