# Supplementary material for: Bio-functionalized magnetic nanoparticles for cost-effective adsorption of U(vi): experimental and theoretical investigation
Source: RSC Adv. 2023 May 16;13(22):15015–23. doi: 10.1039/d3ra00799e (PMC10187032; doi:10.1039/d3ra00799e)
Supplement: RA-013-D3RA00799E-s001 [file RA-013-D3RA00799E-s001.pdf]

Electronic Supplementary Material (ESI) for RSC Advances.
This journal is © The Royal Society of Chemistry 2023

Supplementary information for

Bio-functionalized Magnetic Nanoparticles for Cost-effective Adsorption of U(VI): Experimental and Theoretical Investigation

Chanchal Das[a], Narendra Nath Ghosh[b], Vandana Pulhani[c], Goutam Biswas[a]*, Pallavi Singhal[c]*

[a]*Department of Chemistry, Cooch Behar Panchanan Barma University, Cooch Behar, West Bengal, India 736101*

[b]*Department of Chemistry, University of Gour Banga, Mokdumpur, Malda-732103, India*

[c]*Environmental Monitoring and Assessment Division, Bhabha Atomic Research Centre,Mumbai 400085, India*

***E-mail:** *goutam@cbpbu.ac.in* (Dr. Goutam Biswas)

psinghal@barc.gov.in, *pallavisinghal1987@gmail.com* (Dr. Pallavi Singhal)

Phone : +91-22-2559 2349, Fax: +91-22-2550 5313

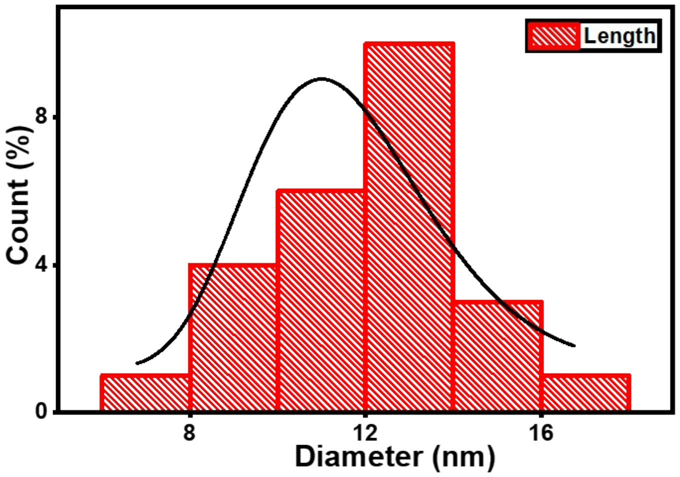

Figure S1: A histogram of size analysis from HRTEM image

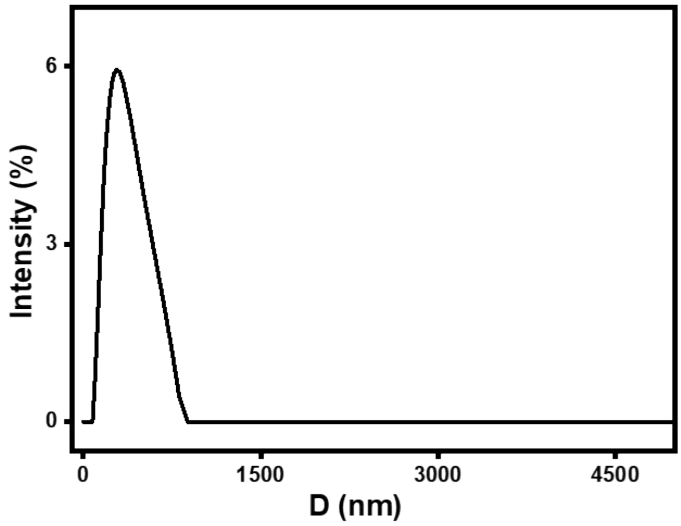

Figure S2: DLS analyzed hydrodynamic size of synthesized CT@MNPs

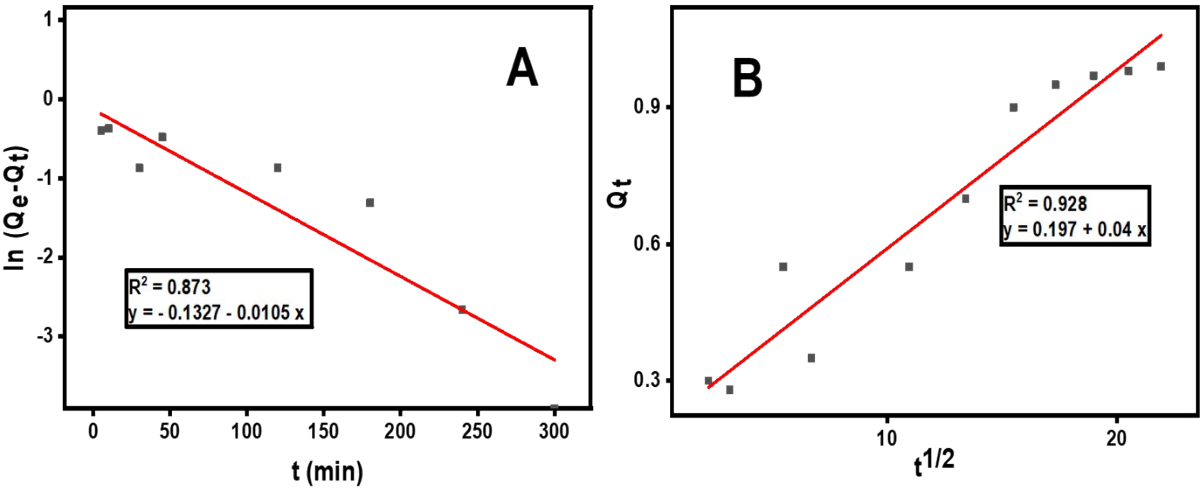

Figure S3: A. Pseudo-first-order kinetic model and B. Intraparticle diffusion model of the U(VI) adsorption onto CT@MNPs.

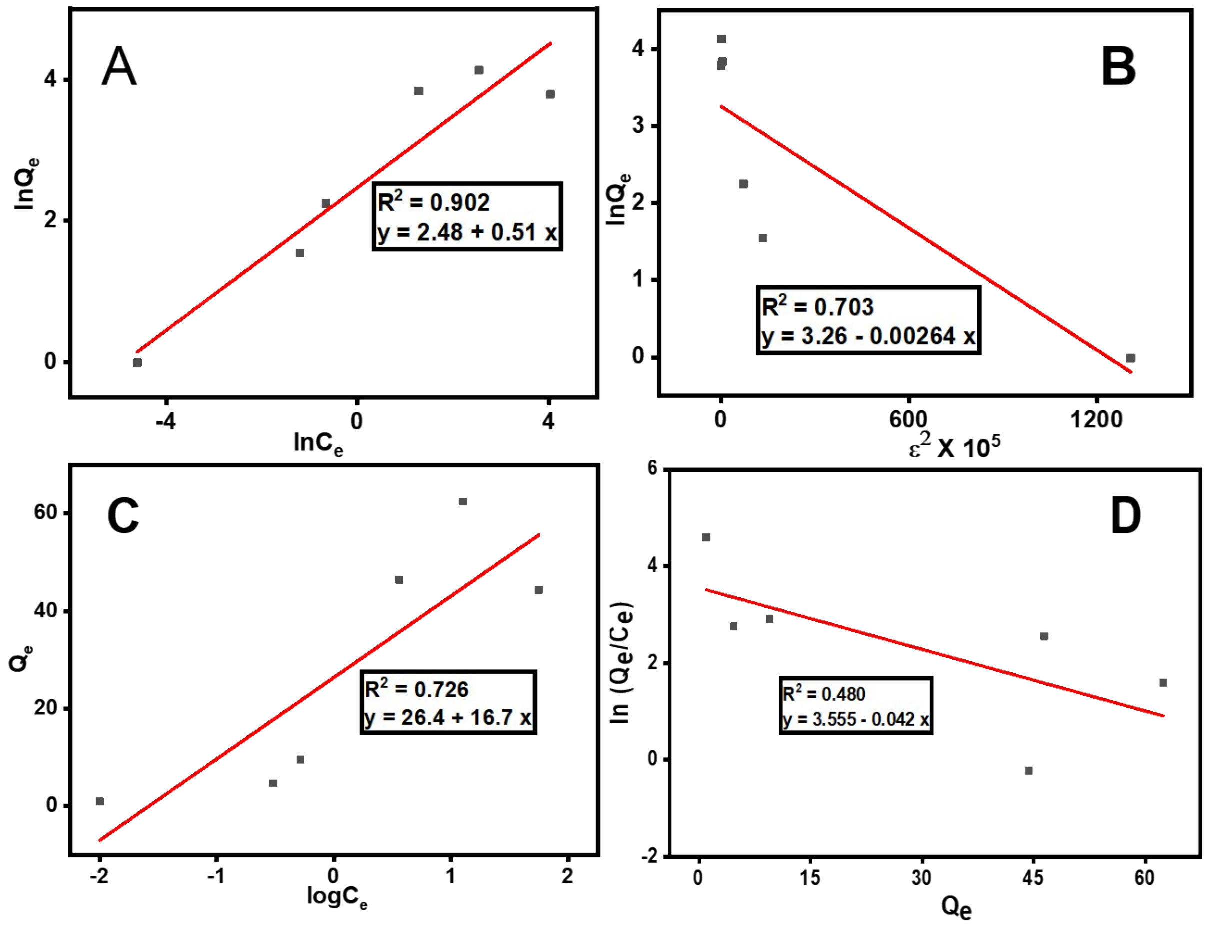

Figure S4: A. Freundlich, B. D-R, C. Temkin and D. Elovich isotherm model of adsorption of U(VI) onto CT@MNPs

Table S1

Various kinetic parameters for the adsorption of U(VI) onto CT@MNPs.

Adsorbate	Concentration (mg L^{-1})	Order	Q_e (exp) (mg g^{-1})	Q_e (cal) (mg g^{-1})	Rate constant*	R^2
U(VI)	1.0	Pseudo-first	0.97	83.4	0.0105	0.873
		Pseudo-second		1.09	0.0160	0.964
		Intraparticle		NA*	0.0400	0.928

	diffusion

* Units for pseudo-first, pseudo-second order and intraparticle diffusion rate constants are min^{-1}, g mg^{-1} min^{-1} and mg g^{-1} $min^{-1/2}$, respectively; * NA = Not applicable.

Table S2

Various isotherm parameters for the adsorption of U(VI) onto CT@MNPs

Langmuir isotherm		
	K_L (L mg^{-1})	2.095
	R_L	0.323
	Q_{max} (mg g^{-1})	45.45
	R^2	0.990
Freundlich isotherm		
	K_F (mg g^{-1})	11.94
	1/n (mg L^{-1})	0.51
	R^2	0.902
Dubinin-Radushkevich (D-R) isotherm		
	β	0.003
	E (kJ mol^{-1})	13.76
	Q_{max} (mg g^{-1})	25.05
	R^2	0.703
Temkin isotherm		
	K_T (L g^{-1})	38.09
	B (J mol^{-1})	341.67
	R^2	0.726
Elovich isotherm		
	K_E	1.47
	Q_{max}	23.81
	R^2	0.480

A comparison table regarding the maximum adsorption capacity is given (Table 3), which tells us that the CT@MNPs was much efficient towards the removal of U(VI).

Table S3

Production cost of CT@MNPs per kg, to use in wastewater purification.

Raw materials	Cost* (USD)	Quantity	Price (US$)
Chemicals			
$FeCl_2$, powder	0.39/kg	0.50 kg	0.19
$FeCl_3$ (anhydrous)	0.24/kg	1.29 kg	0.31
NaOH	0.26/kg	1.28 kg	0.33
Water	0.06/L	100 L	6.00
Cinnamomum tamala leaf	0/kg	5.71 kg	0
Others			
Labor	5.20/employee**	1 day	5.20
Electricity	0.07/kWh	20 kWh	1.40
Maintenance and insurance			0.60
**Total**			**14.03**

* *Price of water and other reagents is based on: IndiaMART (*https://www.indiamart.com*);* ** Employee salary (per day), according to government order: https://wblc.gov.in/synopsys/July/2022.
